# Data-Driven Virtual Sensing for Electrochemical Sensors

**DOI:** 10.3390/s24051396

**Published:** 2024-02-21

**Authors:** Lucia Sangiorgi, Veronica Sberveglieri, Claudio Carnevale, Sabrina De Nardi, Estefanía Nunez-Carmona, Sara Raccagni

**Affiliations:** 1 Department of Mechanical and Industrial Engineering, University of Brescia, 25123 Brescia, Italy; claudio.carnevale@unibs.it (C.C.); sabrina.denardi@unibs.it (S.D.N.); sara.raccagni@unibs.it (S.R.); 2 National Research Council, Institute of Bioscience and Bioresources (CNR-IBBR), Via J.F. Kennedy, 42124 Reggio Emilia, Italy; veronica.sberveglieri@ibbr.cnr.it (V.S.); estefania.nunezcarmona@ibbr.cnr.it (E.N.-C.); 3 Nano Sensor System srl (NASYS), Via Alfonso Catalani, 42124 Reggio Emilia, Italy

**Keywords:** virtual sensing, electrochemical sensors, machine learning

## Abstract

In recent years, the application of machine learning for virtual sensing has revolutionized the monitoring and management of information. In particular, electrochemical sensors generate large amounts of data, allowing the application of complex machine learning/AI models able to (1) reproduce the measured data and (2) predict and manage faults in the measuring sensor. In this work, data-driven models based on an autoregressive model and an artificial neural network have been identified and used to (i) evaluate sensor redundancy and (ii) predict and manage faults in the context of electrochemical sensors for the measurement of ethanol. The approach shows encouraging results in terms of both performance and sensitivity analyses, allowing for the reconstruction of the values measured by two sensors in a series of six sensors with different dopant levels and to reproduce their values after a fault.

## 1. Introduction

In recent decades, the literature has focused on the possibility of indirectly reproducing physical variables through virtual sensors using other correlated measured variables. Thus, virtual sensors incorporate software to enable the computation of a specific variable’s value without direct measurement utilizing data from physically or chemically related measurements [1].

These sensors have proven invaluable in scenarios where placing a physical sensor is unfeasible due to constraints such as inaccessible locations or high costs. Implementing virtual sensors can be approached in two ways:

- In the data-driven approach, time series data of input and output variables are gathered through direct measurements. These data are then utilized to establish a mathematical approximation of the relationship between the measured variables and the sensors’ output [2,3,4]. Machine learning is used in data science to facilitate the identification of patterns and automate the process of data analysis, offering a compelling approach to tackling virtual sensing challenges by leveraging historical data to predict and estimate unmeasured variables due to its capacity to discern complex patterns and relationships within data [5,6,7,8]. Through various algorithms like neural networks, support vector machines, and ensemble methods, machine learning effectively reconstructs and forecasts missing or inaccessible data points [9]. Moreover, machine learning models continuously learn and adapt, refining their predictions over time as they acquire new information. The integration of machine learning into virtual sensing not only enables the estimation of unmeasured variables, but also empowers decision-making processes in various sectors, such as healthcare, manufacturing, and environmental monitoring, resulting in a significant transformation in how we address sensing limitations [10,11,12];
- In the deterministic approach, the physical or chemical connections between input and output variables are leveraged to calculate the unmeasured variable through a virtual sensor [13]. Usually, virtual sensing based on the deterministic approach is performed using methodologies based on the Kalman filter due to its ability to combine available data with system dynamics to estimate unmeasured variables [14,15,16,17]. Its widespread application across various sectors such as autonomous systems, finance, and environmental monitoring highlights its significance in addressing complex problems where direct measurements are unattainable. The Kalman filter stands as a cornerstone for enabling virtual sensing, aiding informed decision making and system optimization.

Thus, the deterministic approach relies on a mathematical model that integrates observed data and system dynamics, allowing precise estimations of unmeasured variables. Data-driven approaches harness an algorithm’s ability to learn from historical data to predict and estimate missing variables, without solely relying on predefined mathematical models. While the deterministic approach focuses on merging observed data and system dynamics, data-driven models identify complex patterns in historical data to predict missing variables, offering greater flexibility in handling nonlinear and intricate situations. Conversely, data-driven modeling requires an initial training phase for existing data, whereas the deterministic approach can be implemented directly using defined mathematical models and available measurements.

For these reasons, the deterministic approach to virtual sensing excels in situations where mathematical models and system dynamics are well understood, offering precise estimations and real-time adaptability. However, it relies heavily on accurate modeling assumptions and struggles with non-linear systems, potentially leading to less accurate estimations in complex scenarios. On the other hand, the advantages of data-driven virtual sensing lie in its adaptability to various scenarios, as no assumptions are required about the system dynamics [18,19]. Yet, it demands substantial data amounts for training, can overfit with insufficient or biased data, and operates as a “black box”, lacking transparency in explaining its predictions.

Chemical sensors such as MOX-type sensors (semiconductor metal oxide gas sensors) have demonstrated the clear advantages of the application of this technology in recent years, and have been used with considerable success in several sectors, ranging from food safety [20], quality control [21] and environmental monitoring to human health, particularly due to their high sensitivity, fast response and low costs. For these reasons, this work investigates the feasibility of integrating electrochemical sensors and virtual sensing in order to benefit from both approaches.

## 2. Materials and Methods

### 2.1. Hardware Setup and Measurements

S3, an acronym for Small Sensor System, is an instrument developed by Nano Sensor Systems Srl (Reggio Emilia, Italy) [22], a start-up affiliated with the University of Brescia. S3 is equipped with an array of chemiresistor MOX-type sensors. The operational mechanisms of this technology exploit the capacity of some metal oxides to become semiconductors when heated to high temperatures (250–400 °C) or when activated by UV light [23]. When the sensing element is activated, a change in the electrical conductance occurs in the sensing material after interaction with the gaseous surrounding environment. The interaction occurring between the oxygen species adsorbed on the surface of the sensitive element and the target molecules present in the gas samples leads to the release of electrons. Subsequently, this electron liberation modulates the electrical properties, including the electrical conductance and resistance [24].

The roughness of the thin film provides a high surface-to-volume ratio and reactivity with gaseous species. Furthermore, the presence of such a very rough surface morphology leads to the high specific area required for high-sensitivity gas sensors [23]. The sensors used in this work were fabricated by the authors using the C920 screen stencil printing machine (Aurel S.p.a., Modigliana, Italy). Sensors were fabricated on a 0.35 mm-thick quartz substrate with pre-deposited heaters and platinum electrical contacts for reading sensor values. The three sensing elements were each printed using screen printing techniques (screen stencil printer C920), employing sensing element pastes previously prepared by the authors. In particular, the sensing elements were composed of SnO_2_, SnO_2_-Au, and SnO_2_-Pd, with a dopant concentration of 0.1% in the last two sensing elements. The addition of the dopants helps to obtain more signal variability and more elasticity regarding the selectivity and sensitivity of the different materials to the surrounding chemical species environment. The sensor response is unspecific, which means that the different sensing elements are affected by different chemical groups in the VOCs present in the environment. The primary sensing material consistently used is SnO_2_. However, by incorporating dopants and adjusting the operating temperature, it is possible to modulate the response of the different sensing elements, thereby achieving a wider spectrum of selectivity and sensitivity.

Following the deposition, a 2 h dying process was conducted to stabilize the sensing elements and facilitate the evaporation of the organic vehicles present in the pastes. Subsequently, the sensors underwent a firing or annealing process, which facilitated the development of a porous nanostructured internal structure. This structure enhanced the permeability of various gases through the sensor. In addition, annealing facilitated the development of a highly durable and resilient crystalline structure, ensuring the long-term stability of the sensors and preventing any potential sensor malfunction. The sensor’s long-term reproducibility was enhanced by this characteristic.

In addition to various semiconductor metal oxide gas sensors, the innovative S3 device is equipped with flow, temperature, and humidity sensors. From the hardware point of view, S3 is composed of three essential parts:

1. Steel sensor chamber: the chamber hosts three MOX sensors separated from the external environment, except for an inlet and an outlet path for the passage of volatile compounds with internal dimensions of 11 cm × 6.5 cm × 1.3 cm.
2. Fluid dynamic circuit: The circuit serves for the distribution of volatile compounds; it is formed by a pump (Knf, model: NMP05B, Nano Sensor Systems Srl, Reggio Emilia, Italy), polyurethane pipes, a solenoid valve, and a metal cylinder which contains activated carbon for filtering any type of odors present outside of the instrument. The pump flow is set by a needle valve positioned at the chamber inlet.
3. Electronics control system: The system records the resistance variations of the sensors, controls their heating, maintains their operating temperature, and facilitates the real-time transmission of the recorded data to the dedicated Web App through an internet connection. This capability enables the storage and analysis of the collected data in the cloud, making S3 an IoT device.

Measurements were performed using a gas cylinder containing 300 ppm of ethanol. Test concentrations were set to 10, 25, and 50 ppm in dry air under varying ambient conditions. A mass flow controller was employed to introduce the test gases, maintaining a total flow rate of 250 sccm. During the measurements, the operational temperature was set to 500 °C. Six different sensors were used in the experiments, each exhibiting differences in the composition of their sensing elements and deposition characteristics, as mentioned earlier.

### 2.2. Data-Driven Models for Virtual Sensing

In this work, two different data-driven approaches have been used for the virtualization of electrochemical sensors: autoregression with exogenous inputs (ARX) model [25] and multi-layer perceptron (MLP) artificial neural networks [26]. In this context, the significance of deploying ARX (autoregression with exogenous inputs) models and artificial neural networks stems from their distinctive capabilities. These models not only provide a means to determine the intricate relationships within electrochemical sensor systems, but also facilitate the representation of complex dynamics and interactions among diverse variables. This aids in a more precise comprehension of sensor behavior.

Furthermore, these models exhibit the ability to capture temporal dependencies and input–output relationships in sensor data, enabling the assessment of redundancy by scrutinizing patterns and correlations among multiple sensors and offering insights into their collective performance and identifying potential overlaps conducive to the creation of virtual sensors.

#### 2.2.1. ARX models

Autoregression with exogenous inputs (ARX) models are a mathematical representation of a dynamic system that encapsulates the relationship between input variables (exogenous variables) and an output variable (endogenous variable) over time [22]. More specifically, these models describe a stochastic process through a linear model, where the output value is linearly dependent on the previous observations. Considering $u{\left(t\right)}^{T}\in R^{n_{ex}}$ and y(t)∈R as the input and output of the system at time t, respectively, the model can be defined by the equation:

(1) $$y\left(t\right)=-\alpha_{1}y\left(t-1\right)-\ldots -\alpha_{n_{a}}y\left(t-n_{a}\right)+\beta_{0}u\left(t-k\right)+\ldots +\beta_{n_{b}}u\left(t-n_{b}-k\right)$$

where:

- $n_{a}$ is the order of the autoregressive part;
- $n_{b}$ is the order of the exogenous part;
- k is the delay between the input and output;
- $\alpha_{i}$ and $\beta_{i}$ are the model coefficients (of the autoregressive and exogenous parts, respectively) to be estimated starting from data.

To achieve a more concise representation, the following vectors $\theta \in R^{n_{a}+n_{b}}$ and $\varphi {\left(t\right)}^{T}\in R^{n_{a}+n_{b}}$ can be introduced:

(2) $$\theta ={\left[\alpha_{1}\;\ldots \;\alpha_{n_{a}}\;\beta_{0}\;\ldots \;\beta_{n_{b}}\right]}^{T}$$

(3) $$\varphi \left(t\right)=\left[-y\left(t-1\right)\;\ldots -y\left(t-n_{a}\right)\;u\left(t-k\right)\;\ldots \;u\left(t-n_{b}-k\right)\right]$$

Thus, Equation (1) can be written as:

(4) y(t)=φ(t) θ

Assuming that the input and output values of a given system have been recorded in n tuples over a time interval $\left[t_{1},\ldots t_{n}\right]$, the parameter vector θ can be estimated using the least squares method:

(5) $$\theta =\left(\Phi^{T}\cdot \Phi^{-1}\right)\cdot \Phi^{T}\cdot Y$$

where:

- $\Phi \left[\begin{array}{ccc} -y\left(t_{1}-1\right)\ldots -y\left(t_{1}-n_{a}\right) & u\left(t_{1}-k\right)\ldots & u\left(t_{1}-n_{b}-k\right)\\ \vdots & \ddots & \vdots \\ -y\left(t_{n}-1\right)\ldots -y\left(t_{n}-n_{a}\right) & u\left(t_{n}-k\right)\ldots & u\left(t_{n}-n_{b}-k\right) \end{array}\right]$ is the matrix of all the model input (including both autoregressive and exogenous parts);
- $Y=\left[\begin{array}{c} y\left(t_{1}\right)\\ \vdots \\ y\left(t_{n}\right) \end{array}\right]$ is the vector of the measured output of the system.

#### 2.2.2. MLP Artificial Neural Networks

In recent years, multi-layer perceptron (MLP) artificial neural networks have emerged as a prominent tool in the field of machine learning, widely used in several tasks for their proficiency in modeling complex data relationships. Therefore, this research project explores the application of neural-network-based models, emphasizing their efficacy in identifying relevant non-linearities within the studied phenomenon, and providing a direct comparison with the linear models employed for this purpose.

The concept underlying artificial neural networks is lightly inspired by the structure and functionality of biological neural networks within the human brain; they consist of interconnected layers of nodes (artificial neurons), where each connection is assigned a specific weight. Assuming an architecture composed of an input layer, a hidden layer with $n_{h}$ neurons, and an output layer with $n_{o}$ neurons, the mathematical formulation of the hidden output $h{\left(t\right)}^{T}\in R^{n_{h}}$ is obtained as:

(6) $$h\left(t\right)=f\left(W_{1}\varphi \left(t\right)+b_{1}\right)$$

where:

- $\varphi {\left(t\right)}^{T}\in R^{n_{a}+n_{b}}$ is the input vector;
- $W_{1}\in R^{n_{h}x\left(n_{a}+n_{b}\right)}$ is the weight matrix connecting the input layer to the hidden layer;
- $b_{1}^{T}\in R^{n_{h}}$ is the bias vector for the hidden layer;
- $f:R^{n_{h}}\to R^{n_{h}}$ is the non-linear activation function for the hidden layer.

Similarly, the final output $y{\left(t\right)}^{T}\in R^{n_{o}}$ is computed as:

(7) $$y\left(t\right)=g\left(W_{2}h\left(t\right)+b_{2}\right)$$

where $W_{2}\in R^{n_{o}x\;n_{h}}$ is the weight matrix connecting the hidden layer to the output layer, $b_{2}^{T}\in R^{n_{o}}$ is the bias vector for the output layer, and $g:R^{n_{o}}\to R^{n_{o}}$ is the non-linear activation function of the output layer.

Therefore, a neural network with the previously defined architecture can be summarized by the equation:

(8) $$y\left(t\right)=g\left(W_{2}f\left(W_{1}\varphi \left(t\right)+b_{1}\right)+b_{2}\right)$$

In this context, the parameters to be estimated during the identification phase are the weights and bias of the network. Typically, the training of these parameters involves executing the backpropagation algorithm on a series of input–output pairs [26].

## 3. Results

### 3.1. Available Data

For the considered tests, the dataset under consideration was derived from a number of measurements obtained from six electrochemical sensors (S1, …, S6) under different conditions. Notably, sensors S1 and S4, S2 and S5, and S3 and S6 are paired and doped with identical materials (SnO_2_, SnO_2_-Au, SnO_2_-Pd, respectively), but were used in different printing processes. Consequently, there is a potential for correlation between the measurements of these paired sensors.

The dataset includes 30 tests performed under varied ambient conditions, with a temperature ranging from 28 to 37 and a relative humidity ranging from 30 to 40. Additionally, the tests involve three levels of ethanol concentration (10 ppm, 25 ppm, and 50 ppm), each lasting for a duration of 4200 s.

### 3.2. Test Definitions

In this study, the virtual sensing approach has been used in two different test cases:

1. Virtual substitution: In this case, a study was performed to select which sensors could be replaced by virtual sensors, using only the measurements produced by the remaining set of sensors. The objective was to reduce the quantity of physical sensors integrated into a device, along with the corresponding costs, by virtualizing sensors with redundant measurements.
2. Virtual switch: In this case, a simulated failure was introduced during the sensor’s operational life, potentially leading to disruptions in sensor readings and the subsequent loss of crucial information. Subsequently, a virtual sensor was employed to reconstruct the unrecorded data from that moment onward.

In each test, two different identification datasets were considered: a smaller one, including only a test performed using the average ethanol concentration under ambient conditions, and a larger one, comprising additional tests to assess the possibility of improving the performance in terms of sensitivity and robustness.

### 3.3. Test Case 1: Virtual Substitution

In this case, the assumption is that the measured value of a sensor $S_{i}$ will be computed using only the data coming from the other sensors. Thus, this means that the resulting models will not contain the autoregressive part:

(9) $$\varphi \left(t\right)=\left[u\left(t-k\right)\;\ldots \;u\left(t-n_{b}-k\right)\right]=\left[u\left(t-k\right)\;\ldots \;u\left(t-n_{b}-k\right)\right]$$

where φ(t) is the vector containing *u*(*t*), i.e., the measurements coming from all the other sensors.

The two models were tested using two main configurations: (i) substitution of only one sensor with its virtual twin using the data from the other five sensors and (ii) substitution of two sensors with their virtual twins using the data coming from the remaining four sensors. In each configuration, different tests were performed to select the value of $n_{b}$ (ranging from 1 to 4), while k was equal to 0 in order to allow the virtual sensor to use the most recent measured data to compute its output. In the first case, the identification dataset including the test case was split into two parts: 70% of the data were used for identification and the remaining 30% were used for validation. For each sensor or couple of sensors, the best model was selected on the basis of the mean absolute error (MAE) on the validation dataset (Table 1 and Table 2).

**Figure 1:**
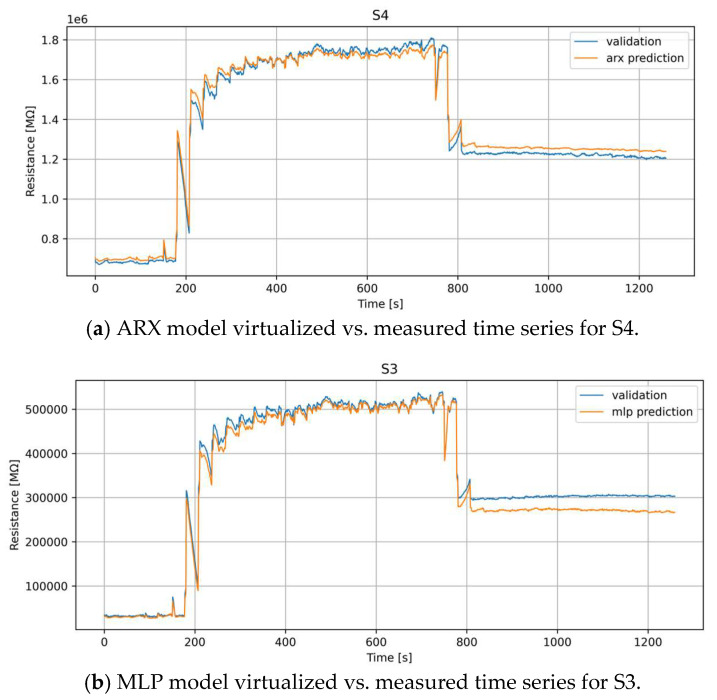
Best sensor selected for virtual substitution by ARX (**a**) and MLP (**b**) models—single substitution.

In the single substitution case, the two models lead to different selections (S4 for ARX and S3 for MLP), with a slightly better performances for ARX than MLP, showing a tendency to better reproduce the dynamical behavior of the data rather than the static response at the end of the dataset (Figure 1).

Table 2 presents the results in terms of the MAE for the double substitution case. In this case, the results of the two models are again different in terms of the sensors selected for virtualization. In this case, the couple S3 and S4 is selected by ARX while the couple S3 and S6 is selected by MLP. This latter result holds significant importance, as S3 and S6, despite being doped with the same material (SnO_2_-Pd), originate from distinct printing processes. Notably, in this scenario, sensors employing diverse doping materials exhibit the capability to replicate the outcomes of sensors utilizing SnO_2_-Pd substrates. This suggests a redundancy in the data derived from SnO_2_-Pd-based sensors in comparison to those with alternative materials, highlighting the robustness and consistency of the former in relation to the latter.

**Figure 2:**
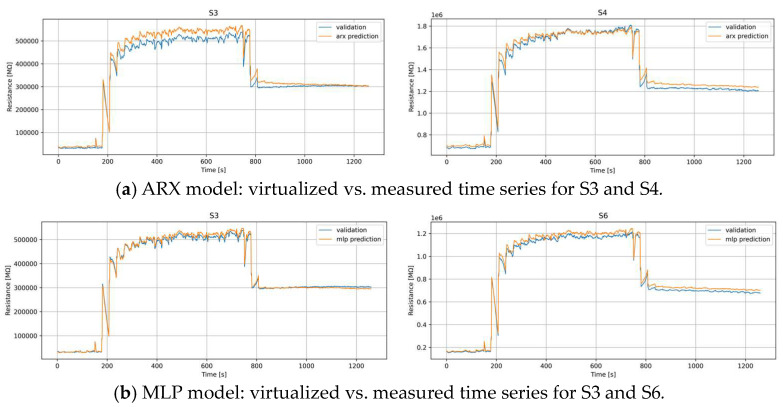
Best sensors selected for virtual substitution by ARX (**a**) and MLP (**b**) models—couple substitution.

Finally, Figure 2 shows how the performance in this case is even better than in the case with single substitution, particularly for MLP, where the bias at the end of the dataset is negligible.

**Figure 3:**
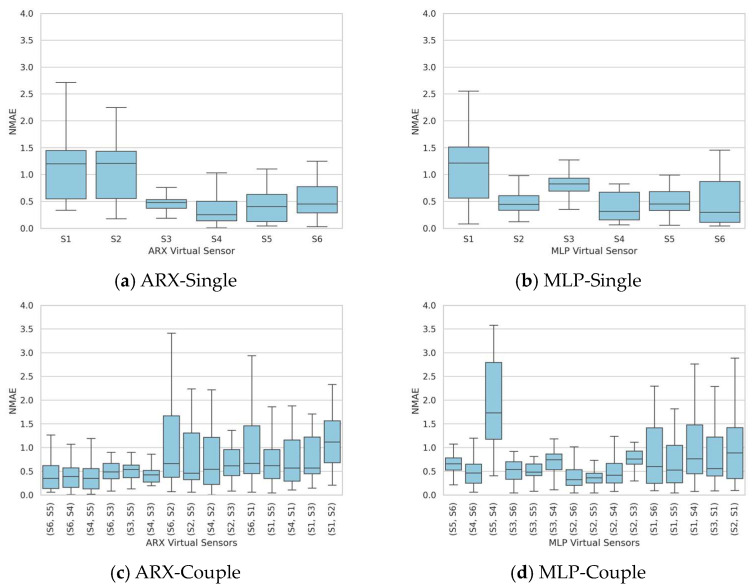
Single-test dataset identification—boxplots of the NMAE series obtained by validating the models on the entire validation dataset of 30 tests for both the single substitution case (**a**,**b**) and the double substitution case (**c**,**d**).

In order to evaluate the sensitivity of the identified sensors to varying concentrations and conditions, the validation of the ARX and MLP model results was performed on the entire dataset, comprising 30 distinct tests. The NMAE was computed for each of these tests. Figure 3 shows the NMAE boxplots for ARX and MLP when the single and double substitutions are performed. The plots show a significant variation in the selected statistical index based on the test configuration, displaying a median of 0.25 and a 75th percentile of 0.5, even in the optimal scenario.

**Figure 4:**
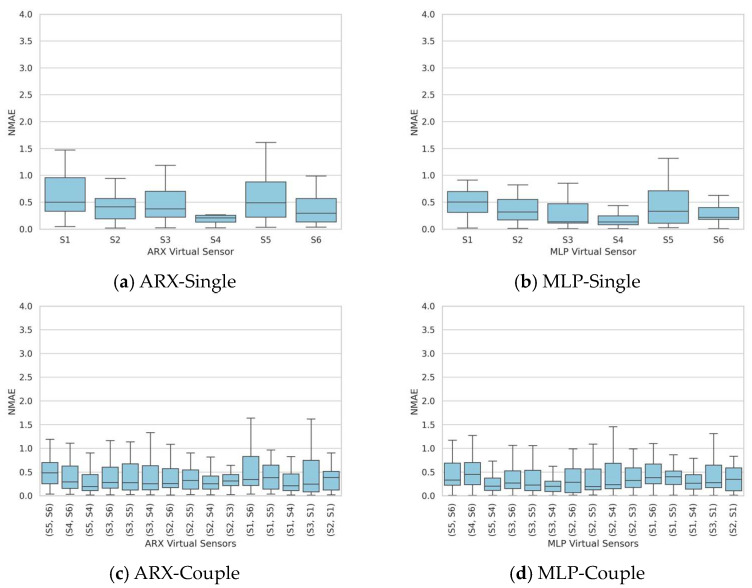
Extended test dataset identification—boxplots of the NMAE series obtained by validating the models on the entire validation dataset of 30 tests for both the single substitution case (**a**,**b**) and the double substitution case (**c**,**d**).

Therefore, to enhance the robustness of the sensor’s performance, an alternative approach was employed: three tests were used for the identification dataset, selected to encompass the different ranges ethanol concentrations, ambient temperatures, and humidities, while the remaining ones were used for the validation dataset. In this configuration, as depicted in Figure 4, the NMAE consistently exhibits a lower variability for each considered sensor, particularly for the top-performing sensors (S4, median of 0.2 and 75th percentile of around 0.25) and the best sensor pairs (the S4 and S5 couple for ARX, with a median of 0.19 and a 75th percentile of 0.5, and the S3 and S4 couple for MLP, with a median of 0.19 and a 75th percentile of 0.2). In this case, the disparity between ARX and MLP is more pronounced for the couple substitution, with a substantial reduction in result variability.

### 3.4. Test Case 2: Sensor Data Recustruction in the Case of Failure

**Figure 5:**
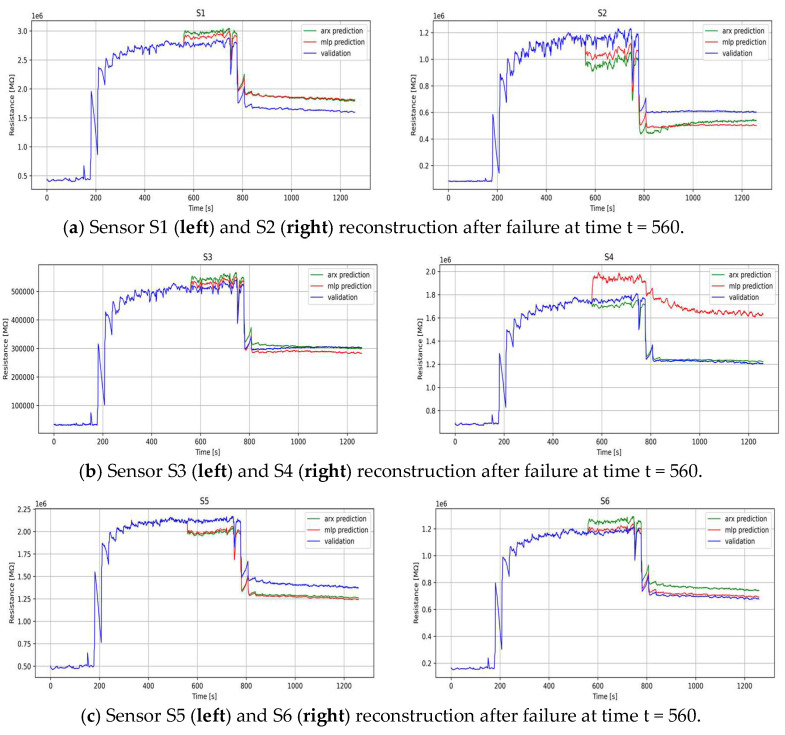
Best sensor selected by ARX (green) and MLP (red) models for virtual reconstruction after failure.

This section shows the results obtained from the reconstruction of missing measurements for the available sensors. The methodology involved the formulation of a virtual model for each sensor, acting as a twin of the sensor itself. In this context, the autoregressive part of the model is considered to work with all the possible available data. Thus, in this context:

(10) $$\varphi \left(t\right)=\left[y\left(t-1\right)\;\ldots \;y\left(t-n_{a}\right)\;u\left(t-k\right)\;\ldots \;u\left(t-n_{b}-k\right)\right]\varphi \left(t\right)=\left[y\left(t-1\right)\;\ldots \;y\left(t-n_{a}\right)\;u\left(t-k\right)\;\ldots \;u\left(t-n_{b}-k\right)\right]$$

for Equations (4) and (8). The models were constructed by varying both the autoregressive and exogenous components, denoted as $n_{a}\;\mathrm{and}\;n_{b}$, spanning orders from 1 to 4, and the best model for each sensor was selected based on the MAE. After model identification, the reconstruction of missing measurements was executed for the validation segment, assuming that a failure occurs from time *t* = 560 to the end of the dataset. After the reconstruction, the model’s accuracy was evaluated by analyzing its predictive capacity when missing measurements are detected. Figure 5 depicts the reconstructions derived from the two distinct models, ARX and MLP, applied individually to each sensor and compared with the previously validated dataset. A visual analysis of the figures reveals the notable efficacy of both models in accurately predicting the missing data, particularly in scenarios where interruptions occur in the extrapolated data, often induced by sensor faults. It is imperative to underscore that, overall, the MLP model exhibits a superior performance in comparison to the ARX model, with the noteworthy exception observed in the case of sensor S4.

**Figure 6:**
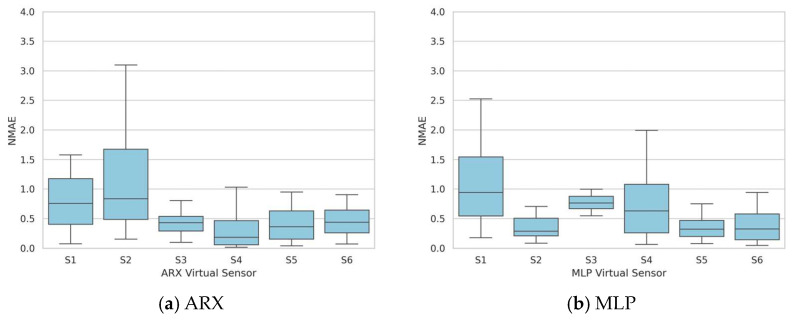
Single-test dataset identification—boxplots of the NMAE series obtained by validating the models on the entire validation dataset of 30 tests.

Once again, the entire collection of 30 experiments was employed to validate the ARX and MLP models and to evaluate the sensitivity of the identified sensors to varying concentrations and conditions. The NMAE was computed for each test, and the corresponding boxplots are displayed in Figure 6. The graphs reveal substantial fluctuations in the statistical index based on the validation test employed, with certain instances showing median values exceeding 0.5.

**Figure 7:**
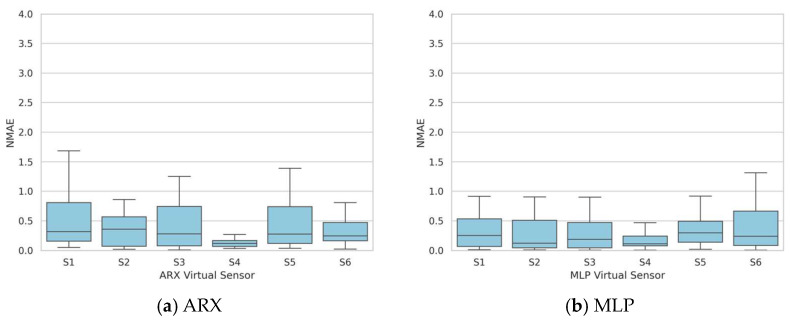
Extended test dataset identification—boxplots of the NMAE series obtained by validating the models on the entire validation dataset of 30 tests.

Thus, to improve the robustness of the sensors, the same approach as in the previous section has been implemented here. Three tests were utilized for model identification, and the remaining ones were allocated for validation. Figure 7 presents the obtained NMAE distribution in terms of a boxplot, showing a limited variability in the sensor performances and a median value consistently below 0.5. Notably, S4 demonstrates a superior performance compared to the other sensors, whether the measurements are reconstructed using the ARX model (median of 0.12 and 75th percentile of approximately 0.2) or the MLP model (median of 0.11 and 75th percentile of approximately 0.25).

## 4. Conclusions

In this work, the application of virtual sensing techniques based on machine learning has been investigated for electrochemical sensors. Different tests have been performed for ethanol measurements using state-of-the-art sensors developed by NASYS, a start-up affiliated with the University of Brescia. Two different data-driven models have been used to evaluate (i) the redundancy among sensors implemented on the same board and (ii) the possibility of reconstructing the measurement of the sensors in case of a fault. The selected models are based on an autoregression with exogenous inputs (ARX) model and an multi-layer perceptron (MLP) artificial neural network. The results are really encouraging, with both models showing good performances in both situations. Future developments may involve testing the S3 sensors under wet conditions in order to further enhance the sensitivity and reproducibility of the entire chain (hardware and virtual sensors). Additionally, there is potential for a smooth integration between machine learning and these types of sensors to extend the application to forecasting measurements over an extended time horizon. This aims to facilitate the use of electrochemical sensors in more sophisticated control schemes, such as model predictive control of systems.

## Figures and Tables

**Table 1:** MAE for the single-sensor substitution case for ARX and MLP (the selected best model for virtualization is in bold).

Sensor	MAE ARX	MAE MLP
S1	0.096	0.063
S2	0.153	0.155
S3	0.054	**0.046**
S4	**0.019**	0.173
S5	0.075	0.070
S6	0.078	0.049

**Table 2:** MAE for the double-sensor substitution case for ARX and MLP (the selected best model for virtualization is in bold).

Sensors	Average MAE ARX	Average MAE MLP
S1, S2	0.146	0.099
S1, S3	0.071	0.057
S1, S4	0.069	0.103
S1, S5	0.046	0.055
S1, S6	0.096	0.101
S2, S3	0.109	0.156
S2, S4	0.085	0.151
S2, S5	0.068	0.064
S2, S6	0.068	0.063
S3, S4	**0.038**	0.109
S3, S5	0.055	0.047
S3, S6	0.060	**0.024**
S4, S5	0.046	0.110
S4, S6	0.049	0.091
S5, S6	0.044	0.045

## Data Availability

Data are contained within the article.
